# 192 Behavioural and observational analysis of ‘Be Active with Arthritis’ exercise programme to promote and maintain physical activity (PA) in people with arthritis

**DOI:** 10.1093/eurpub/ckae114.044

**Published:** 2024-09-26

**Authors:** Aoife Stephenson, Maia Harris, Rameen Khalid, Deirdre Hurley-Osing, David French, Suzanne McDonough

**Affiliations:** Royal College of Surgeons in Ireland, Dublin, Ireland; Royal College of Surgeons in Ireland, Dublin, Ireland; Royal College of Surgeons in Ireland, Dublin, Ireland; University College Dublin, Ireland; University of Manchester, UK; Royal College of Surgeons in Ireland, Dublin, Ireland

## Abstract

**Purpose:**

The Irish Society of Chartered Physiotherapists (ISCP) and Arthritis Ireland (AI) developed ‘Be Active with Arthritis’ (BAWA), a physiotherapy-led, group-based exercise programme to promote PA in people with arthritis. Participant feedback suggests BAWA participants initially become more active, but many reduce PA levels after programme completion. This may be explained by the absence of behaviour change techniques (BCTs) supporting PA maintenance.

The study objectives are:

1. Identify core BCTs recommended in BAWA training materials and distinguish between those used for supporting in-class exercise versus those supporting day-to-day PA maintenance.

2. Complete an observational analysis of BAWA delivery to explore which BCTs are used to support PA maintenance.

**Methods:**

Training materials for the delivering therapists were reviewed for the presence of core BCTs. The core BCTs linked to PA in-class and PA maintenance were identified. Team members attended and audio-recorded BAWA classes; recordings were analysed for the presence of core BCTs (in-class and maintenance).

**Results:**

Twenty-three core PA in-class BCTs (23/93), and 11 core PA maintenance BCTs were identified in the training materials. Researchers observed 13 BAWA classes from four programme blocks; led by three different therapists, at three separate locations. Seven of the 11 core maintenance BCTs (64%) were identified at least once from the recordings. The most frequently used were: “Generalisation of target behaviour”, “Credible source” and “Body changes”. An additional seven BCTs were identified in the recordings and documented as potentially pertinent to PA maintenance. The most frequently used additional BCTs were “Behavioural practice/rehearsal” and “Habit formation”.

**Conclusions:**

The inclusion of BCTs into the training material was low. There was limited prevalence of BCTs previously thought to be linked to PA maintenance. The observed absence of BCTs such as “Self-monitoring of behaviour” in daily life using devices such as smartwatches was notable. Future development of a maintenance intervention for people living with arthritis should adopt evidence based BCTs and involve people with arthritis in making judgements on acceptability.

**Support/funding source:**

HRCI/HRB in collaboration with AI, HRCI-HRB-2022-010.

